# Supplementary material for: Chromothripsis during telomere crisis is independent of NHEJ, and consistent with a replicative origin
Source: Genome Res. 2019 May;29(5):737–49. doi: 10.1101/gr.240705.118 (PMC6499312; doi:10.1101/gr.240705.118)
Supplement: Supplemental Material [file supp_gr.240705.118_Supplemental_file_1.zip › contigs/annotated_contigs/DB92/contig.2.DB92_length_336_mean_cov_5.80357142857.docx]

**DB92_length_336_mean_cov_5.80357142857**

```
AGGTAGCTTGAAATTAGCCATAGTGGGAATATTTATATCATGGAAATTGACAAATGCTGAAAATCAGGGCTTATTTTCAGAGTTAGCTT
 >chr8:69394843-69395017 - E=2e-90
ACTGGCATGCCACTGAGTATAAAGAGTAATGTTAAAGAAAATACCTGTGTAAGTAAAACTTTTATCAATAAATGGGTGATCA|ACA|TT
                                                                                   >chr
AGCATCTCTACTCCAACAGTTCTTTGATCCTGTGGGGCCTCCAATCTGTTGACTTACAGGATTTATGTACAATCCGTCATCCTCCTGTA
3:60931722-60931887 - E=6e-85
TCCTCACTTTCCTCCTTGCTCAGCTCAGATGCTATGGTTCATCTCTTCTTTACATGCACACTGAACTCCTT
```
